# Fibril-Forming Motifs Are Essential and Sufficient for the Fibrillization of Human Tau

**DOI:** 10.1371/journal.pone.0038903

**Published:** 2012-06-11

**Authors:** Sheng-Rong Meng, Ying-Zhu Zhu, Tong Guo, Xiao-Ling Liu, Jie Chen, Yi Liang

**Affiliations:** State Key Laboratory of Virology, College of Life Sciences, Wuhan University, Wuhan, China; University Medical Center Groningen, The Netherlands

## Abstract

**Background:**

The misfolding of amyloidogenic proteins including human Tau protein, human prion protein, and human α-synuclein is involved in neurodegenerative diseases such as Alzheimer disease, prion disease, and Parkinson disease. Although a lot of research on such amyloidogenic proteins has been done, we do not know the determinants that drive these proteins to form fibrils and thereby induce neurodegenerative diseases. In this study, we want to know the role of fibril-forming motifs from such amyloidogenic proteins in the fibrillization of human Tau protein.

**Methodology/Principal Findings:**

As evidenced by thioflavin T binding and turbidity assays, transmission electron microscopy, and circular dichroism, fibril-forming motifs are essential and sufficient for the fibrillization of microtubule-associated protein Tau: only when both of its fibril-forming motifs, PHF6 and PHF6*, are deleted can recombinant human Tau fragment Tau_244–372_ lose its ability to form fibrils, and the insertion of unrelated fibril-forming motifs from other amyloidogenic proteins, such as human prion protein, yeast prion protein, human α-synuclein, and human amyloid β, into the disabled Tau protein can retrieve its ability to form fibrils. Furthermore, this retrieval is independent of the insertion location on Tau_244–372_.

**Conclusions/Significance:**

We demonstrate for the first time that insertion of fibril-forming motifs can replace PHF6/PHF6* motifs, driving human Tau protein to form fibrils with different morphologies and different kinetic parameters. Our results suggest that fibril-forming motifs play a key role in the fibrillization of human Tau protein and could be the determinants of amyloidogenic proteins tending to misfold, thereby causing the initiation and development of neurodegenerative diseases. Our study also touches on the importance of amyloid “strains”: changes to the amyloidgenic driver region results in altered structural morphologies at the macromolecular level.

## Introduction

The abnormal aggregation of proteins plays an important role in the functions of proteins: the misfolding of amyloidogenic proteins can cause serious neurodegenerative diseases, such as human Tau protein and human amyloid β peptide in Alzheimer disease, human α-synuclein in Parkinson disease, human polyglutamine-containing peptides in Huntington disease, and human/bovine prion proteins in prion diseases [1]–[7]; some are helpful for organisms to survive in environmental threats, for example, Sup35 in yeast; and some are required for the normal functions of the organisms [7], such as curlin in *E. coli* [7], [8], Pmel17 in the pigmentation of mammals [9], and many peptide or protein hormones are stored in the form of amyloid fibrils [10].

Actually, the potential of misfolding of proteins are influenced by many factors: abnormal cellular environments, including aberrant ion concentrations [11], [12] and unbalanced oxidative stress [13]; covalent modification of proteins, such as the hyperphosphorylation of Tau protein [13], [14] and aged glycation of β_2_-microglobulin [15], [16]; crowded physiological environments [14], [17]; and pathogenic mutations in amyloidogenic proteins which enable or promote their ability of aggregation [13], [18]. However, according to Anfinsen’s dogma [19] the primary structures of proteins may be the determinants of the potential of aggregation of these proteins such as human Tau protein. Human Tau can form fibrils without any posttranslational modifications and any pathogenic mutations *in vitro* [12], [20], [21], but mutation of the amino acid sequences does influence the kinetics of Tau filament formation or even make the fibrillization impossible [22], [23].

Although a lot of research on such amyloidogenic proteins has been done, we do not know the determinants that drive these proteins to form fibrils and thereby induce neurodegenerative diseases. Using methods such as NMR [24], proline-scanning [25], and positive fibrillization assays [26], [27], scientists have identified some fibril-forming motifs from the reported amyloid proteins. These fibril-forming motifs can form amyloid fibrils and microcrystals *in vitro*, and X-ray structures of these microcrystals reveal a dry, tightly self-complementing steric zipper architecture model [28], [29]. Based on these data, researchers try to find determinants of these proteins through bioinformatics methods [30]–[33]. A algorithm based on these structures has been developed, and by using this algorithm, the fibril-forming motifs are characterized as peptides with their Rosetta energy below the threshold of -23 kcal/mol [34], [35]. A systematic genome-wide survey on *S. cerevisiae* reveals that the enrichment of asparagines rather than glutamines, and the spacing of prolines and charged amino acids contribute to the aggregation of proteins [36].

Human microtubule-associated protein Tau is a natively unfolded protein in solution [3], [37]. Filamentous Tau has been shown to be the main component of neurofibrillary tangles, a pathological hallmark of Alzheimer disease [3], [22], [37]–[40]. Two fibril-forming motifs ^275^VQIINK^280^ (PHF6*) and ^306^VQIVYK^311^ (PHF6) are very important for the fibrillization of Tau protein: the fibrillization of a truncated fragment of Tau PHF43 requires the existence of PHF6 [22]; mutations occurring in any of these fibril-forming motifs will abrogate the ability of polymerization of the truncated Tau protein [41]. Tau_244–372_, the core fragment of human Tau protein, is a frequently used model for Tau fibrillization, can form fibrils with the help of heparin *in vitro* in a relatively short time [12], [20], [21].

In this study, we want to know the role of fibril-forming motifs in the fibrillization of human Tau protein. We investigated the potential primary structure determinants of filament formation of human Tau protein by using several biophysical methods, such as assays based on thioflavin T (ThT) binding and turbidity, transmission electron microscopy (TEM), and far-UV circular dichroism (CD). We demonstrated for the first time that insertion of unrelated fibril-forming motifs from other amyloidogenic proteins, such as human prion protein, yeast prion protein, human α-synuclein, and human amyloid β, could replace PHF6/PHF6* motifs of human Tau protein, driving Tau_244–372_ to form fibrils with different morphologies and different kinetic parameters.

## Materials and Methods

### Ethics Statement

All research involving original human work was approved by the Institutional Review Board of the College of Life Sciences, Wuhan University (Wuhan, China), leaded by Dr. Hong-Bing Shu, the Dean of the college, in accordance with the guidelines for the protection of human subjects. Written informed consent for the original human work that produced the plasmid samples was obtained.

### Materials

Heparin (average molecular mass of 6 kDa) and ThT were from Sigma-Aldrich (St. Louis, MO). Dithiothreitol (DTT) was obtained from Amresco (Solon, OH). DNA polymerase Kod-plus was from Takara (Tokyo, Japan). Other chemicals used were made in China and of analytical grade.

### Plasmids and Proteins

The construction of plasmid expressing Tau_244–372_ was carried as described [12]. The following primers for human Tau mutants were synthesized, for example, P1, ACTGCCGCCTCCCGGGACGTGTTTGATATTATCC, P2, CAGCAGCAGCAGC AGCAGCCAGTTGACCTGAGCAAGGTGACCTCCAAGTGTGG, P3, CTGCTG CTGCTGCTGCTGACTGCCGCCTCCCGGGACGTGTTTGATATTATCC, and P4, CCAGTTGACCTGAGCAAGGTGACCTCCAAGTGTGG were designed to substitute VQIVYK of Tau_244–372_ with QQQQQQ. 0.5 µl Kod-Plus (1 U/µl), 2.5 µl 10× Kod-Plus Buffer, 2.5 µl of 2 mM dNTP, 1 µl of 25 mM MgSO_4_, 1 µl plasmid (about 50 ng/µl), 1 µl P1 (10 pmol/µl), and 1 µl P2 (10 pmol/µl) were added into Tube A, and then added Mini-Q water into adjust the total volume to 25 µl. 0.5 µl Kod-Plus (1 U/µl), 2.5 µl 10× Kod-Plus Buffer, 2.5 µl of 2 mM dNTP, 1 µl of 25 mM MgSO_4_, 1 µl plasmid (about 50 ng/µl), 1 µl P3 (10 pmol/µl), and 1 µl P4 (10 pmol/µl) were added into Tube B, and then added Mini-Q water into adjust the total volume to 25 µl. The following PCR program was run for tubes A and B: Step 1: 94°C for 2 min; Step 2: 94°C for 15 s; and Step 3: 68°C for 1 min/kb plasmid. Steps 2 and 3 were repeated for 25 times. Tubes A and B were mixed, and the following program was run: Step 1: 98°C for 2 min; and Step 2: 40°C for 1 min. Steps 1 and 2 were repeated for 3 times. The quantity of the product was checked with 1% agarose gel and the product was digested with DpnI. Plasmids containing target sequences were transformed into *Escherichia coli* BL21 DE3 strain.

The expression of recombinant human Tau fragment Tau_244–372_ and its mutants were induced with 400 µM isopropyl-β-D-thiogalactopyranoside and cultured for 3 h. Cell pellets of 2 liter culture were collected and re-suspended in 100 ml buffer A (20 mM phosphate buffer containing 2 mM DTT, pH 7.0) and then sonicated at 200 W for 30 min. 500 mM NaCl was added into the mixture and then the mixture was boiled at 100°C for 15 min. After centrifugation at 17,000 *g* for 30 min at 4°C, supernatant was collected and dialyzed against buffer A extensively. The sample was then loaded onto a SP sepharose column (20-ml bead volume) and washed with 400 ml buffer A. The target protein was obtained by washing the column using 500 ml of 20 mM phosphate buffer containing 2 mM DTT and 0–400 mM NaCl. Tau fragment was then concentrated and dialyzed against 50 mM Tris-HCl buffer containing 2 mM DTT (pH 7.5) extensively, and then stored at −80°C. Purified Tau protein was analyzed by SDS-PAGE with one band and confirmed by mass spectrometry. The concentration of human Tau fragment was determined according to its absorbance at 214 nm with a standard calibration curve drawn by bovine serum albumin.

### Fibrillization of Proteins

The fibrillization for Tau_244–372_ and its mutants was in a mixture of 8 µM Tau protein, 2 µM heparin, and 1 mM DTT in 50 mM Tris-HCl buffer (pH 7.5) at 37°C for at least 10 h for ThT binding assays and TEM experiments, or in a mixture of 20 µM Tau protein, 20 µM heparin, and 1 mM DTT in 20 mM NaH_2_PO_4_-Na_2_HPO_4_ buffer (pH 7.4) at 37°C for up to 24 h for far-UV CD measurements, ThT binding assays, and TEM experiments.

### Thioflavin T Binding Assays

A 2.5 mM ThT stock solution freshly prepared in 50 mM Tris-HCl buffer (pH 7.5) was added into the fibrillization system of Tau protein, giving a final concentration of 16/40 µM. The kinetics was monitored in 96-well plates at 37°C in SpectraMax M2 microplate reader (Molecular Devices, Sunnyvale, CA) using excitation at 440 nm and emission at 480 nm with a wavelength cut off at 475 nm, or in an LS-55 luminescence spectrometer (PerkinElmer Life Sciences, Shelton, CT) using excitation at 440 nm and emission at 480 nm. Each sample was run in triplicate.

### Turbidity Assays

The fibrillization for Tau_244–372_ and its mutants was in a mixture of 50 µM Tau protein, 12.5 µM heparin, and 1 mM DTT in 50 mM Tris-HCl buffer (pH 7.5) at 37°C for up to 15 h for turbidity assays. Turbidity at 350 nm was monitored in 96-well plates at 37°C in SpectraMax M2 microplate reader (Molecular Devices, Sunnyvale, CA). Each sample was run in triplicate.

### Transmission Electron Microscopy

The formation of fibrils by Tau_244–372_ and its mutants was confirmed by electron microscopy of negatively stained samples. Sample aliquots of 10 µl were placed on carbon-coated copper grids (Shanghai Mainstream Trading Company, Shanghai, China), and left at room temperature for 1–2 min, rinsed with H_2_O twice, and then stained with 2% (w/v) uranyl acetate for another 1–2 min. The stained samples were examined using an H-8100 transmission electron microscope (Hitachi, Tokyo, Japan) operating at 100 kV or an FEI Tecnai G2 20 transmission electron microscope (Hillsboro, OR) operating at 200 kV.

### Far-UV CD Measurements

Under standard conditions, 20 µM Tau protein was incubated in 20 mM NaH_2_PO_4_-Na_2_HPO_4_ buffer (pH 7.4) containing 1 mM DTT and 20 µM heparin at 37°C for up to 24 h. Circular dichroism spectra were obtained by using a Jasco J-810 spectropolarimeter (Jasco Corp., Tokyo, Japan) with a thermostated cell holder. Quartz cell with a 1 mm light-path was used for measurements in the far-UV region. Spectra were recorded from 195 to 250 nm for far-UV CD. The final concentration of Tau protein for far-UV CD measurements was kept at 10 µM so that the high tension voltage associated to CD spectra was less than 600 V. The spectra of all scans were corrected relative to the buffer blank. The mean residue molar ellipticity [θ] (deg⋅ cm^2^⋅ dmol^−1^) was calculated using the formula 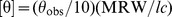, where *θ*_obs_ is the observed ellipticity in deg, MRW the mean residue molecular weight (106.1 Daltons for Tau fragment), *l* the path length in cm, and *c* the protein concentration in g/ml.

## Results

### A Ligase-independent Plasmid Mutation Method

**Figure 1:**
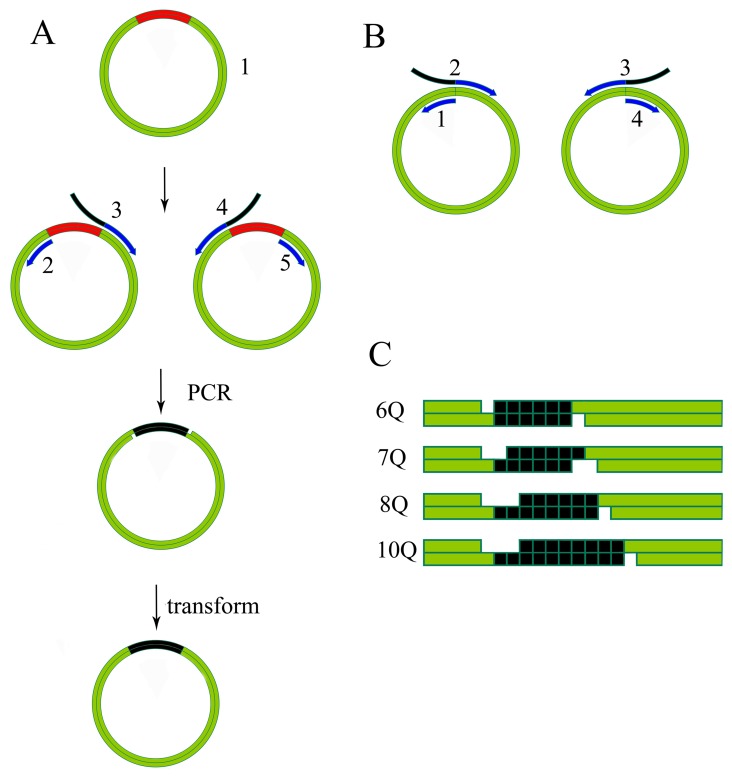
Design of primers and protocol of PCR are based on the following new strategy. This is a ligase-independent plasmid mutation method. (A) Substitution of a relatively long fragment of DNA in a plasmid with another one. To substitute the DNA fragment marked with red color with another DNA fragment marked with black, two pair of primers were designed. The first pair (2 and 3) were used to generate linear double strand DNA molecular (dsDNA) with the target fragment at the 3′ end of the sensitive strand and the second pair (4 and 5) were used to generate linear dsDNA with the target fragment at the 5′ end of the sensitive strand. (B) Primers for insertion of a DNA fragment to a plasmid. To get an insertion of special DNA fragment indicated with black color into a plasmid, the two pairs of primers (1–4) are designed to amplify the whole plasmid to linear dsDNA with the target at the 5′ or 3′ end. (C) Some unique cases of mutagenesis. We also got some unexpected results while try to constructs new mutagenesis. We designed primers to insert six glutamines (Q) into the target location of our gene, and besides the excepted products of 6Q-insertion, we also get some 7Q-insertions. This, however, indicates that the products are formed in the range of our plan. Based on this phenomenon, we designed primers to generate PCR products that are not complementary completely with each other and we get the 8Q-insertion and 10Q-insertion successfully.

The sequences and source of unrelated fibril-forming motifs inserted are listed in Table 1. Design of primers and protocol of PCR are based on our new strategy, and the details are shown in Fig. 1 and the corresponding figure legend. All of the mutants were confirmed by DNA sequencing. Our sequencing results indicated that this ligase-independent plasmid mutation method dramatically increased the efficiency of short-fragment substitution by transforming the classical blunt-end ligation into sticky end-ligation, compared with the traditional QuickChange PCR method.

**Table 1:** Source of fibril-forming motifs in our experiments.

Fragment	Source
SNQNNF	Human prion protein [29]
NNQQNY	Yeast prion Sup35 [28], [29]
QQQQQQ	Yeast prion SUP35 and human huntingtin [29]
GVATVA	Human α-synuclein [29], [42]
GGVVIA	Human amyloid β [29]
IFQINS	Human lysozyme [29], [34]
NHVTLS	Human β_2_-microglobulin [27], [29]
SQAIIH	Myoglobin [29], [34]
GGGGGG	Negative control reported [42]
FERQHM	Negative control from RNase A [35]

### Fibril-forming Motifs are Essential for the Fibrillization of Tau_244–372_

**Figure 2:**
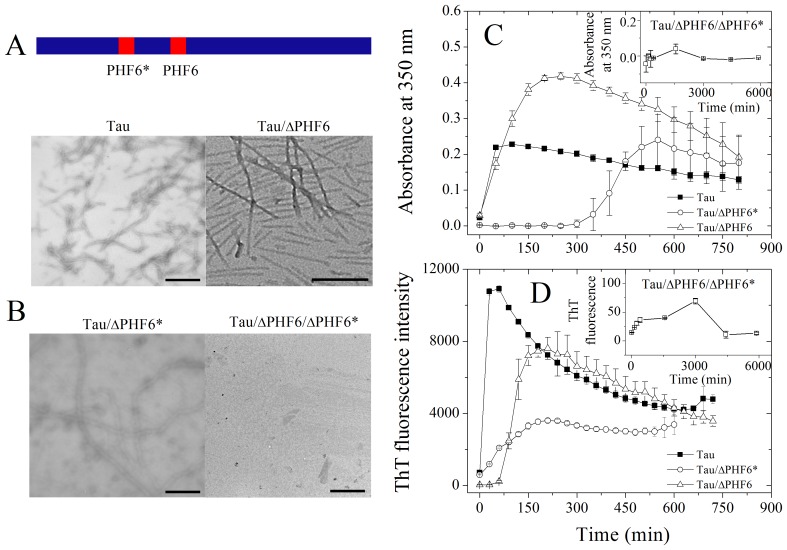
Fibril-forming motifs are essential for the fibrillization of Tau_244–372_. (A) Location of the two fibril-forming motifs ^275^VQIINK^280^ (PHF6*) and ^306^VQIVYK^311^ (PHF6) at Tau_244–372_: VQIINK is in the R2 part of Tau_244–372_, and VQIVYK is in the R3 part of Tau_244–372_. (B) Negative-stain transmission electron micrographs of Tau_244–372_, Tau_244–372_/ΔPHF6, Tau_244–372_/ΔPHF6*, and Tau_244–372_/ΔPHF6/ΔPHF6* (scale bar 400 nm). (**C**) Kinetic curves for the aggregation of Tau_244–372_, Tau_244–372_/ΔPHF6, Tau_244–372_/ΔPHF6*, and Tau_244–372_/ΔPHF6/ΔPHF6*, monitored by the turbidity at 350 nm. The concentration of Tau protein was 50 µM, and 50 mM Tris-HCl buffer (pH 7.5) containing 1 mM DTT and 12.5 µM heparin was used. The assays were carried out at 37°C. Tau_244–372_, Tau_244–372_/ΔPHF6, and Tau_244–372_/ΔPHF6* finished their progress of aggregation in a short time of observation (15 h), but in a relatively long time of observation (100 h) Tau_244–372_/ΔPHF6/ΔPHF6* did not show any clue of aggregation (the inset of C). (D) Kinetic curves for the aggregation of Tau_244–372_, Tau_244–372_/ΔPHF6, Tau_244–372_/ΔPHF6*, and Tau_244–372_/ΔPHF6/ΔPHF6*, monitored by ThT fluorescence. The long time incubation of Tau_244–372_/ΔPHF6/ΔPHF6* is shown in the inset of D. The concentration of Tau protein was 8 µM, and 50 mM Tris-HCl buffer (pH 7.5) containing 1 mM DTT and 2 µM heparin was used. The assays were carried out at 37°C. The observation time was 12 h for Tau_244–372_, Tau_244–372_ΔPHF6, and Tau_244–372_ΔPHF6*, and 100 h for Tau_244–372_/ΔPHF6/ΔPHF6*.

It has been reported that PHF6 and PHF6* are both very important for the fibrillization of human Tau protein but only PHF6 is essential for filament formation [23]. Research on PHF43, containing PHF6 but not PHF6* shows that, PHF6 is essential for the fibrillization of that truncated peptides [22]. Three mutants of Tau_244–372_, Tau_244–372_/ΔPHF6, Tau_244–372_/ΔPHF6*, and Tau_244–372_/ΔPHF6/ΔPHF6* (Fig. 2A), were thus constructed, expressed, and purified. We investigated fibril formation of wild-type Tau_244–372_ and its three mutants by using ThT binding assays, turbidity assays, TEM, and far-UV CD. During a relatively long time of observation (100 h), Tau_244–372_/ΔPHF6/ΔPHF6* failed to form fibrils (Fig. 2B and the inset of Fig. 2C and 2D). The aggregation of wild-type Tau_244–372_ was the most rapid process among these processes, and the kinetic curves reached the maximum within about 1 h (Fig. 2C and 2D). The deletion of one of the fibril-forming motifs influenced the kinetics of Tau fibrillization markedly. As shown in Fig. 2C and 2D, the lag time of either Tau_244–372_/ΔPHF6 or Tau_244–372_/ΔPHF6* was remarkably longer than that of Tau_244–372_, however, they finally proved their ability of fibrillization. This is strong evidence showing that both of the fibril-forming motifs are required for the fibrillization of Tau_244–372_, and each of them can enable fibril formation of Tau_244–372_. Because only when both of its fibril-forming motifs, PHF6 and PHF6*, were deleted could recombinant human Tau fragment Tau_244–372_ lose its ability to form fibrils, fibril-forming motifs are essential for the fibrillization of human Tau protein.

**Figure 3:**
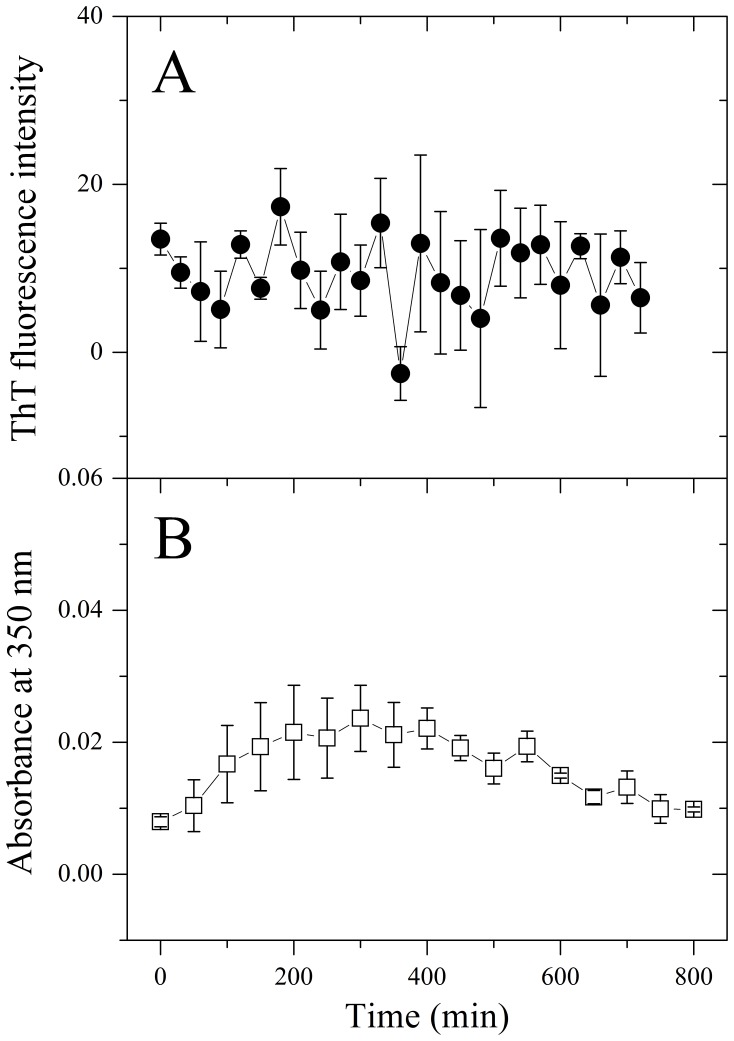
Tau_244–372_/ΔPHF6*/PHF6^306^GSRSRT does not aggregate on the investigated time scale. (A) Kinetic curves for the aggregation of Tau_244–372_/ΔPHF6*/PHF6^306^GSRSRT, monitored by ThT fluorescence. The concentration of Tau protein was 8 µM, and 50 mM Tris-HCl buffer (pH 7.5) containing 1 mM DTT and 2 µM heparin was used. The assays were carried out at 37°C, and the observation time was 12 h. (B) Kinetic curves for the aggregation of Tau_244–372_/ΔPHF6*/PHF6^306^GSRSRT, monitored by the turbidity at 350 nm. The concentration of Tau protein was 50 µM, and 50 mM Tris-HCl buffer (pH 7.5) containing 1 mM DTT and 12.5 µM heparin was used. The assays were carried out at 37°C, and the observation time was 15 h.

To exclude the possibility that it is the deletion itself (the shorted length of the protein) but not the fibril-forming motifs involved contributes to the abrogation of fibrillization, we constructed another mutation Tau_244–372_/ΔPHF6*/PHF6^306^GSRSRT through inserting a hexapeptide GSRSRT (Tau_207–212_), which does not have the property of the fibril-forming motifs [42], into Tau_244–372_/ΔPHF6* at the location of PHF6, as a negative control. As shown in Fig. 3, such a mutant failed to aggregate on the investigated time scale.

Our results suggest that the interaction of the two fibril-forming motifs is not required for the fibrillization of Tau_244–372_ and that both motifs perform their fibril-forming functions independently.

### Insertion of Fibril-forming Motifs Replaces PHF6/PHF6* Motifs, Driving Tau_244–372_ to Form Fibrils with Different Morphologies and Different Kinetic Parameters

**Figure 4:**
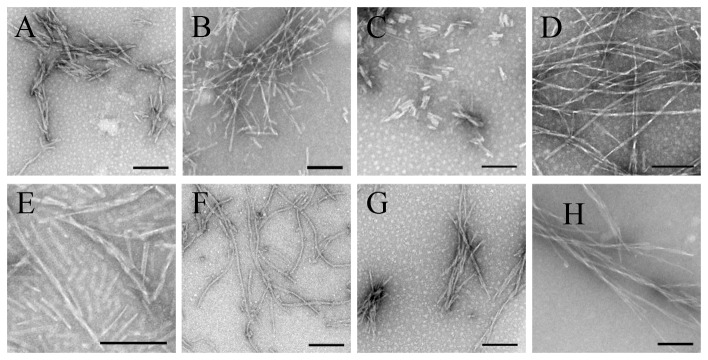
Insertion of fibril-forming motifs from other amyloidogenic proteins into the disabled Tau protein can retrieve its ability to form fibrils−TEM measurements. Negative-stain transmission electron micrographs of the following mutants: insertion of SNQNNF (A), NNQQNY (B), QQQQQQ (C), GVATVA (D), GGVVIA (E), IFQINS (F), NHVTLS (G), and SQAIIH (H) into Tau_244–372_/ΔPHF6/ΔPHF6* at the location of PHF6 after incubation for 24 h. All the scale bars were 200 nm.

**Figure 5:**
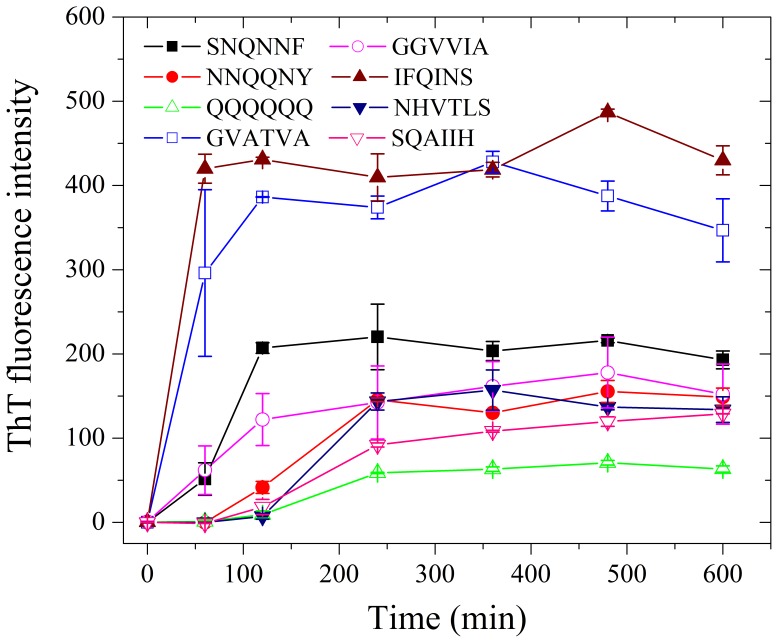
Insertion of fibril-forming motifs from other amyloidogenic proteins into the disabled Tau protein can retrieve its ability to form fibrils−ThT binding assays. Kinetic curves for the aggregation of Tau_244–372_/ΔPHF6/ΔPHF6* inserted by SNQNNF (black), NNQQNY (red), QQQQQQ (green), GVATVA (blue), GGVVIA (magenta), IFQINS (wine), NHVTLS (navy), and SQAIIH (pink), monitored by ThT fluorescence. The concentration of Tau protein was 20 µM, and 20 mM NaH_2_PO_4_-Na_2_HPO_4_ buffer (pH 7.4) containing 1 mM DTT and 20 µM heparin was used. The assays were carried out at 37°C, and the observation time was 10 h.

To determine whether other fibril-forming motifs can replace PHF6/PHF6* motifs, we inserted fibril-forming motifs from other amyloidogenic proteins, such as human prion protein, yeast prion protein, human α-synuclein, and human amyloid β [27]–[29], [34], [42], into the disabled Tau_244–372_/ΔPHF6/ΔPHF6*. Such fibril-forming motifs are obtained from references indiscriminately, and the detailed sequences and their sources are shown in Table 1. These fibril-forming motifs can form fibrils or microcrystals *in vitro*, and the crystal structures of some of them, such as SNQNNF and NNQQNY, have been determined [27]–[29], [34], [42]. Figs. 4 and 5 representatively show negative-stain transmission electron micrographs and kinetic curves for the aggregation of the following mutants: insertion of SNQNNF, NNQQNY, QQQQQQ, GVATVA, GGVVIA, IFQINS, NHVTLS, and SQAIIH, into Tau_244–372_/ΔPHF6/ΔPHF6* at the location of PHF6. As evidenced by ThT binding assays and TEM, although these fibril-forming motifs come from different amyloidogenic proteins, all of them did drive the disabled Tau protein to form fibrils under moderate conditions (Figs. 4 and 5). As shown in Figs. 4 and 5, although the fibril-forming motifs only make up of about 5% of the amino acid sequences of these mutants, fibrils formed from such different Tau mutants were of different morphologies and different kinetic parameters. For example, insertion of GVATVA, a fibril-forming motif from human α-synuclein [29], [42], into Tau_244–372_/ΔPHF6/ΔPHF6* at the location of PHF6 produced long and branched fibrils (Fig. 4D) with shorter lag time and higher ThT fluorescence intensity (Fig. 5), but insertion of QQQQQQ, a fibril-forming motif from yeast prion SUP35 and human huntingtin [30], into Tau_244–372_/ΔPHF6/ΔPHF6* produced short amyloid fibrils (Fig. 4C) with much longer lag time and much lower ThT fluorescence intensity (Fig. 5). Because the insertion of unrelated fibril-forming motifs from other amyloidogenic proteins into the disabled Tau protein could retrieve its ability to form fibrils, fibril-forming motifs are sufficient for the fibrillization of human Tau protein.

**Figure 6:**
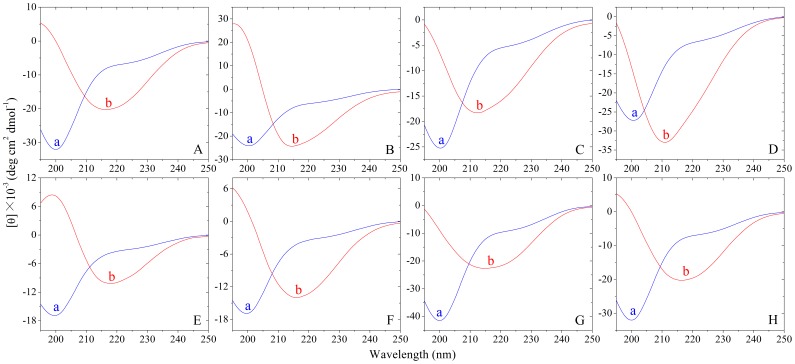
Insertion of fibril-forming motifs from other amyloidogenic proteins into the disabled Tau protein can retrieve its ability to form fibrils−CD measurements. Far-UV CD spectra of the following mutants: insertion of SNQNNF (A), NNQQNY (B), QQQQQQ (C), GVATVA (D), GGVVIA (E), IFQINS (F), NHVTLS (G), and SQAIIH (H) into Tau_244–372_/ΔPHF6/ΔPHF6* at the location of PHF6. Curve a: native Tau protein. Curve b: filaments produced from Tau protein after incubation for 24 h. The CD signals have no signs of high tension voltage saturation and all of the curves here have been smoothed.

CD spectroscopy was used to further determine whether other fibril-forming motifs can replace PHF6/PHF6* motifs. Fig. 6 shows the CD spectra of native Tau mutants and filaments produced by Tau mutants. As shown in Fig. 6, at the beginning, the CD spectra measured for all of Tau mutants had a strong negative peak at 200 nm, indicative of a largely random coil structure. After incubation for 24 h, a single minimum around 216 nm was observed for most of Tau mutant samples (Fig. 6A–6C and 6E–6H), which is typical of predominant β-sheet structure and a characteristic for filament formation. After incubation for 24 h, however, a single minimum around 211 nm (but not 216 nm) was observed for fibril sample of Tau_244–372_/ΔPHF6/ΔPHF6* inserted by GVATVA (Fig. 6D), indicating that fibrils formed by such a Tau mutant contained less β-sheet structure than those formed by other Tau mutants.

**Figure 7:**
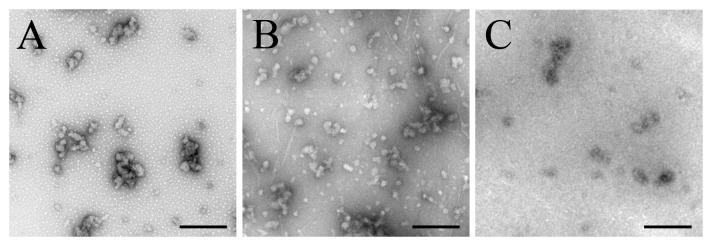
GGGGGG and FERQHM did not induce Tau filament formation−TEM measurements. Negative-stain transmission electron micrographs of the following mutants: insertion of GGGGGG (B) and FERQHM (C), two hexapetides predicted to have no ability to aggregate, into Tau_244–372_/ΔPHF6/ΔPHF6* (A) at the location of PHF6 after incubation for 24 h. All the scale bars were 200 nm.

**Figure 8:**
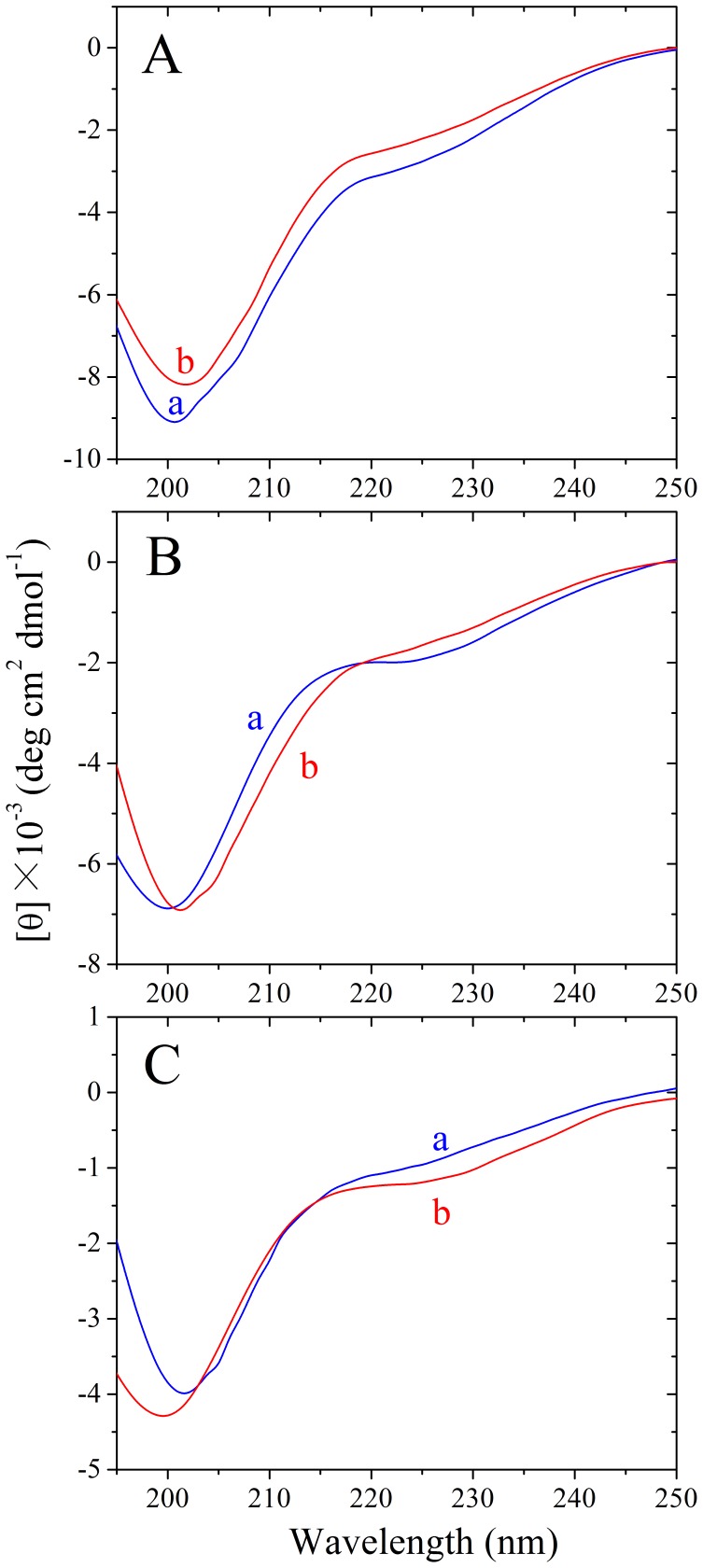
GGGGGG and FERQHM did not induce Tau filament formation−CD measurements. Far-UV CD spectra of the following mutants: insertion of GGGGGG (B) and FERQHM (C) into Tau_244–372_/ΔPHF6/ΔPHF6* (A) at the location of PHF6. Curve a: native Tau protein. Curve b: Tau protein after incubation with heparin and DTT at 37°C for 24 h. The CD signals have no signs of high tension voltage saturation and all of the curves here have been smoothed.

We then inserted GGGGGG and FERQHM, two hexapetides predicted to have no ability to aggregate [35], [42], into Tau_244–372_/ΔPHF6/ΔPHF6* at the location of PHF6. As revealed by TEM (Fig. 7) and CD spectroscopy (Fig. 8), GGGGGG and FERQHM did not induce Tau filament formation on the investigated time scale of 24 h. Our negative control experiments verified that insertion of non-fibril forming peptides could not drive the disabled Tau protein to form amyloid fibrils (Figs. 3, 7, and 8).

Clearly, insertion of fibril-forming motifs from other amyloidogenic proteins, such as human prion protein, yeast prion protein, human α-synuclein, and human amyloid β, could replace PHF6/PHF6* motifs of human Tau protein, driving Tau_244–372_ to form fibrils with different morphologies and different kinetic parameters.

### The Retrieval is Independent of the Insertion Location on Tau_244–372_

**Figure 9:**
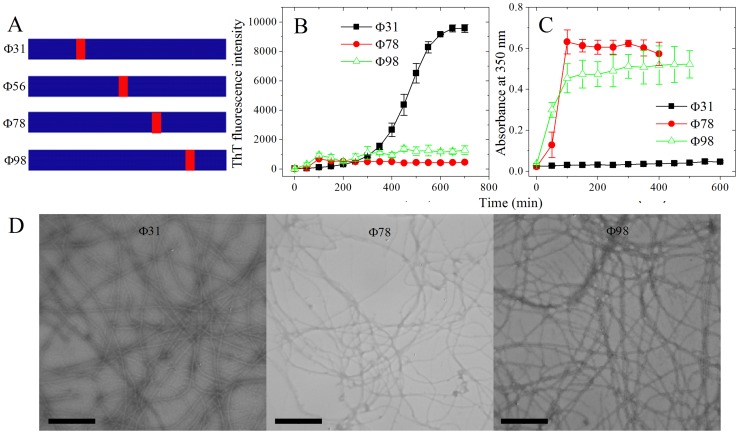
The retrieval is independent of the location of the insertion on Tau_244–372_. (A) Four insertions of IFQINS into different locations. Φ31, Φ56, Φ78, and Φ98 represent Tau mutants in which IFQINS was inserted between 30th and 31th, 55th and 56th, 77th and 78th, and 97th and 98th amino acids of Tau_244–372_ respectively. Φ56 is the same as the mutant inserted by IFQINS at the location of PHF6, and its negative-stain transmission electron micrograph is shown in Figure 4. (B) Kinetic curves for the aggregation of Φ31, Φ78, and Φ98, monitored by ThT fluorescence. The concentration of Tau protein was 8 µM, and 50 mM Tris-HCl buffer (pH 7.5) containing 1 mM DTT and 2 µM heparin was used. The assays were carried out at 37°C, and the observation time was 12 h. (C) Kinetic curves for the aggregation of Φ31, Φ78, and Φ98, monitored by the turbidity at 350 nm. The concentration of Tau protein was 50 µM, and 50 mM Tris-HCl buffer (pH 7.5) containing 1 mM DTT and 12.5 µM heparin was used. The assays were carried out at 37°C. (D) Negative-stain transmission electron micrographs of Φ31, Φ78, and Φ98 (scale bar 400 nm) after incubation for 12 h.

It has been reported that fibril-forming motifs can drive a non-fibrillizing protein RNase A to the amyloid state, and the insertion is located at C-terminal hinge loop, although it is long known that RNase A is capable of forming domain-swapped oligomers [42]. The insertion location may supply the possibility for fibril-forming motifs to exposed to the outside of the protein and thereby the interaction between fibril-forming motifs happens, which is important for protein aggregation [34]. To determine whether this retrieval depends on the insertion location, we inserted one of the fibril-forming motifs, IFQINS, into Tau_244–372_/ΔPHF6/ΔPHF6* at four different locations and obtained four mutants, Φ31, Φ56, Φ78, and Φ98 (Fig. 9A). These locations were chosen randomly. The results from ThT binding and turbidity assays and TEM showed that all four mutants had the ability to form fibrils (Fig. 9B–9D and Fig. 4), indicating that the retrieval of fibrillization function is independent of the insertion location of fibril-forming motifs on Tau_244–372_.

### Impact of the Length of the Fibril-forming Motifs on Tau_244–372_ Fibrillization Kinetics

Mutants generated from the insertion of a different fibril-forming motif into Tau_244–372_/ΔPHF6/ΔPHF6* had different kinetic parameters (Fig. 5). We obtained kinetic parameters for Tau mutants by fitting the experimental data to the empirical Hill equation [14], [21]:
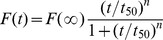
(1)Here *F*(∞) is the fluorescence intensity in the long time limit, *t*_50_ is the elapsed time at which *F* is equal to one-half of *F*(∞), and *n* is a cooperativity parameter.

The length of polyQ has been demonstrated to be involved in fibrillization kinetics of intracellular huntingtin aggregate formation [43], [44]. We thus constructed a series of mutants inserted by different number of glutamines into Tau_244–372_/ΔPHF6/ΔPHF6*. Data from ThT binding assays of these mutants were fitted to Eq. 1 and three kinetic parameters were obtained and compared. As shown in Table 2, the number of glutamines inserted into Tau_244–372_/ΔPHF6/ΔPHF6* had different effects on fibrillization kinetic parameters for these Tau mutants. *F*(∞) increased remarkably with the number of glutamines inserted and then decreased to some extent with the number of glutamines larger than 7, and *n* increased noticeably with the length of polyQ, while *t*_50_ had no significant correlation with the number of glutamines. Our data demonstrated that the length of the fibril-forming motifs is involved in fibrillization kinetics of Tau mutants.

**Table 2:** Kinetic parameters for the growth of ThT fluorescence intensity of mutants inserted by different number of glutamines into Tau_244–372_/ΔPHF6/ΔPHF6*.

	*F*(∞)	*t*_50_ (min)	*n*
6Q	102.4±3.6	5.9±0.3	2.5±0.2
7Q	183.5±5.6	5.8±0.2	5.2±0.6
8Q	69.4±5.4	7.6±0.4	5.7±1.5
10Q	81.9±4.3	5.6±0.3	8.7±3.7

Best-fit values of these kinetic parameters were derived from non-linear least squares modeling of the Hill equation to the experimental data. The concentration of Tau protein was 20 µM, and 20 mM NaH_2_PO_4_-Na_2_HPO_4_ buffer (pH 7.4) containing 1 mM DTT and 20 µM heparin was used. The assays were carried out at 37°C, and the observation time was 24 h. The ± sign is a standard deviation.

## Discussion

In the present study, we perform all fibrillization experiments in the presence of the inducer heparin, not only for wild-type Tau_244–372_ and deletion mutants, but also for insertion variants. It is known that poly-anions such as heparin trigger fibrillization of human Tau protein [12], [20]–[23], [41]. Therefore it would be interesting to explore whether these insertion mutants no longer require heparin for aggregation, since these sequences themselves are generally able to assemble in the absence of heparin [29]. Our additional experiments verified that only one of the eight insertion mutants can form fibrils in the absence of heparin but the others can not form fibrils in the absence of heparin on the investigated time scale of 14 days (Figs. S1 and S2). As shown in Figs. S1 and S2F, insertion of IFQINS, a fibril-forming motif from human lysozyme [29], [34], into Tau_244–372_/ΔPHF6/ΔPHF6* at the location of PHF6 produced straight filaments in the absence of heparin (Fig. S2F) with much longer lag time and remarkable lower ThT fluorescence intensity (Fig. S1), compared with those in the presence of heparin (Fig. 5). It has been reported that the binding of heparin to Tau monomer induces conformational changes in Tau, as well as reducing the large net positive charge borne by Tau protein, thus reducing the activation energy required to add Tau to the end of a growing Tau fiber and accelerating fibril growth [20]. Therefore, it is possible that the Tau sequence outside PHF6/PHF6* motifs relies on presence of heparin for assembly and that it is due to fibril-forming motifs to balance negative effects of the ‘non’-amyloidogenic regions. The fibril-forming ability of IFQINS is so strong that such an insertion mutant no longer requires heparin for aggregation.

The absorbance traces of all the forms in Fig. 2 converge to a signal of about 0.2. The same happens in ThT traces. A possible explanation for this behavior has been proposed. Fibrils formed by wild-type Tau_244–372_ and its two single deletion mutants are probably loose and of more β-sheet structure during the middle time period. At the final stage, however, the fibrils will become more compact and some β-sheet structure will be buried, leading to reduced turbidity and reduced accessibility of ThT-binding sites.

It should be pointed out that different fibrillization conditions were used in this work. As shown in Figs. 2 and S3 (8 µM Tau protein, 2 µM heparin, and Tris-HCl buffer) and Figs. 4, 5, 6, 7, 8 (20 µM Tau protein, 20 µM heparin, and phosphate buffer), the change of fibrillization conditions does not affect the phenomena we are observing that fibril-forming motifs are essential and sufficient for the fibrillization of human Tau, although such conditions are usually associated to changes in the aggregation rate of human Tau [20] and possibly morphology.

Fibril-forming motifs found from amyloidogenic proteins have been reported to be important for the misfolding of these proteins: they can form fibrils *in vitro*, and the damage of their ability to form β-sheet *via* site-directed substitution with proline will disturb or even erase the misfolding of these proteins composing them [34], [42]. The present study demonstrated that insertion of fibril-forming motifs from other amyloidogenic proteins, such as human prion protein and human α-synuclein, could replace PHF6/PHF6* motifs of human Tau protein, driving Tau_244–372_ to form fibrils, but insertion of non-fibril forming peptides could not replace PHF6/PHF6* motifs, unable to drive Tau_244–372_ to form fibrils. Furthermore, fibrils produced by such Tau mutants were of different morphologies and different kinetic parameters, although the fibril-forming motifs only make up of about 5% of the amino acid sequences of these mutants. The data all supports the view that PHF6 and 6* regions are important for aggregation and that deletion of both regions abrogates assembly. The insertion of fibril-forming motifs from other amyloidogenic proteins rescues assembly ability in the double deletion mutant, although results in altered morphologies. Our study impacts on the importance of sequence for amyloidogenesis and also touches on the importance of amyloid “strains”: changes to the amyloidgenic driver region results in altered structural morphologies at the macromolecular level. Because these changes in morphology may reflect the structures of the short peptides alone [29] and different fibril morphologies may have different underlying molecular structures [45], [46], it would be worthwhile to extend the current work to investigate whether each “driver” peptide can propagate a particular morphology. These are planned for the future. Our results here suggest that fibril-forming motifs play a key role in the fibrillization of human Tau protein and could be the determinants of amyloidogenic proteins tending to misfold, thereby causing the initiation and development of neurodegenerative diseases. Thus it is a possible to slow down, stop, or even clear the misfolding of amyloidogenic proteins *via* blocking their fibril-forming motifs by drugs. In fact, fibril-forming motif LVEALYL from insulin has been successfully used to block the fibrillization of insulin [47]. Such a fibril-forming motif can form complementary steric zipper structure with LVEALYL in insulin, and thus shield the space and suppress the fibrillization of insulin [47]. Using known atomic structures of fibril-forming motifs as templates, the Eisenberg lab has recently designed and characterized an all-D-amino-acid inhibitor of the fibrillization of Tau protein [48], and has presented atomic structures of fibril-forming motifs of proteins involved in Alzheimer disease in complex with small molecule binders [49]. We also tried to block the fibrillization of human Tau with one of its fibril-forming motifs VQIVYK, but unfortunately failed to block the fibrillization of human Tau. This, however, suggests that, we should try to find or design more effective components besides using their own fibril-forming motifs in the future.

Although amyloid fibrils can cause many serious diseases of human being, they are very interesting and useful state of proteins. Amyloids are very stable, as they are acid resistant, alkali resistant, and protease resistant, and can exist for a relative long time [7]–[10]. Amyloids can drive macromolecules into a super high local concentrations and such an enrichment will have a dramatic effect, and it has been reported that amyloid formed by Sup35p can increase the sensitivity of immunoassay up to 100-fold with protein G and methyl-parathion hydrolase attached with it [50].

Our results make it possible that fibril-forming motifs could be fused into proteins including enzymes, antibodies, and structural proteins and change their structural stability, sensitivity, capacity to resist extreme environments, and other functions at nanometer level. In this study we find that fibril-forming motifs are of different capacity to drive fibril formation: some of them, for example IFQINS from human lysozyme [29], [34], if inserted in the proper locations, they will form beautiful fibrils with higher intensity of ThT fluorescence and lower turbidity (Figs. 4F, 5, and 9B–9D). In the next step, we will try to fuse fibril-forming motifs to some proteins and test the change of their functions.

In conclusion we focus on the role of fibril-forming motifs in the fibrillization of human Tau, and have shown that: (i) deletion of both PHF and PHF6* hexapeptide motifs eliminates fibrillization propensity of human Tau; (ii) insertion of unrelated fibril-forming motifs from other amyloidogenic proteins, such as human prion protein and human α-synuclein, can replace PHF6/PHF6* motifs of human Tau, driving Tau_244–372_ to form fibrils with different morphologies and different kinetic parameters; (iii) insertion of non-fibril forming peptides can not replace PHF6/PHF6* motifs; (iv) the retrieval of fibrillization function does not depend on the insertion location of fibril-forming motifs on human Tau; (v) fibril-forming motifs are essential and sufficient for the fibrillization of human Tau. Information obtained from the present study can enhance our understanding of the molecular mechanisms of neurodegenerative diseases such as Alzheimer disease and prion disease, and should lead to a better understanding of how proteins misfold and how proteins avoid misfolding in physiological environments.

## Supporting Information

Figure S1 **One of the eight insertion mutants can form fibrils in the absence of heparin** **but the others can not−ThT binding assays.** Kinetic curves for the aggregation of Tau_244–372_/ΔPHF6/ΔPHF6* inserted by SNQNNF (black), NNQQNY (red), QQQQQQ (green), GVATVA (blue), GGVVIA (magenta), IFQINS (wine), NHVTLS (navy), and SQAIIH (pink) incubated in the absence of heparin, monitored by ThT fluorescence. The concentration of Tau protein was 20 µM, and 20 mM NaH_2_PO_4_-Na_2_HPO_4_ buffer (pH 7.4) containing 1 mM DTT was used. The assays were carried out at 37°C, and the observation time was 14 days.
(DOC)

Figure S2 **One of the eight insertion mutants can form fibrils in the absence of heparin** **but the others can not−TEM measurements.** Negative-stain transmission electron micrographs of the following eight mutants: insertion of SNQNNF (A), NNQQNY (B), QQQQQQ (C), GVATVA (D), GGVVIA (E), IFQINS (F), NHVTLS (G), and SQAIIH (H) into Tau_244–372_/ΔPHF6/ΔPHF6* at the location of PHF6 after incubation for 14 days in the absence of heparin. Amyloid fibrils were clearly observed (F). All the scale bars were 200 nm.
(DOC)

Figure S3 **Insertion of fibril-forming motifs from other amyloidogenic proteins into the disabled Tau protein can retrieve its ability to form fibrils−ThT binding assays.** Kinetic curves for the aggregation of Tau_244–372_/ΔPHF6/ΔPHF6* inserted by SNQNNF (black), NNQQNY (red), QQQQQQ (green), GVATVA (blue), GGVVIA (magenta), IFQINS (wine), NHVTLS (navy), and SQAIIH (pink), monitored by ThT fluorescence. The concentration of Tau protein was 8 µM, and 50 mM Tris-HCl buffer (pH 7.5) containing 1 mM DTT and 2 µM heparin was used. The assays were carried out at 37°C, and the observation time was 10 h.
(DOC)
