# Supplementary figures and images for: Fibril-Forming Motifs Are Essential and Sufficient for the Fibrillization of Human Tau
Source: PLoS One. 2012 Jun 11;7(6):e38903. doi: 10.1371/journal.pone.0038903 (PMC3372541; doi:10.1371/journal.pone.0038903)

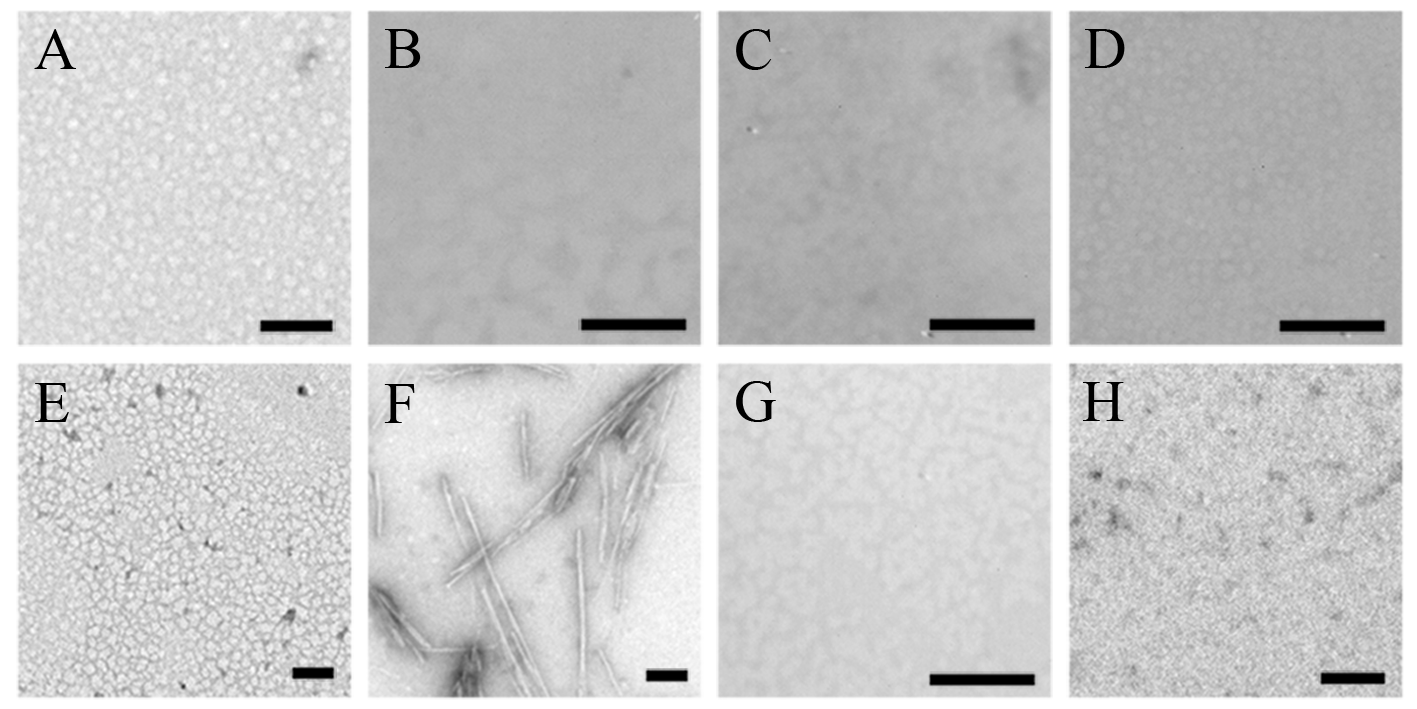

Supplement: Figure S2 — One of the eight insertion mutants can form fibrils in the absence of heparin but the others can not−TEM measurements. Negative-stain transmission electron micrographs of the following eight mutants: insertion of SNQNNF (A), NNQQNY (B), QQQQQQ (C), GVATVA (D), GGVVIA (E), IFQINS (F), NHVTLS (G), and SQAIIH (H) into Tau244–372/ΔPHF6/ΔPHF6* at the location of PHF6 after incubation for 14 days in the absence of heparin. Amyloid fibrils were clearly observed (F). All the scale bars were 200 nm. (DOC) [file pone.0038903.s002.doc]
