# Supplementary material for: Fibril-Forming Motifs Are Essential and Sufficient for the Fibrillization of Human Tau
Source: PLoS One. 2012 Jun 11;7(6):e38903. doi: 10.1371/journal.pone.0038903 (PMC3372541; doi:10.1371/journal.pone.0038903)
Supplement: Supplementary file 2 [file pone.0038903.s001.doc]

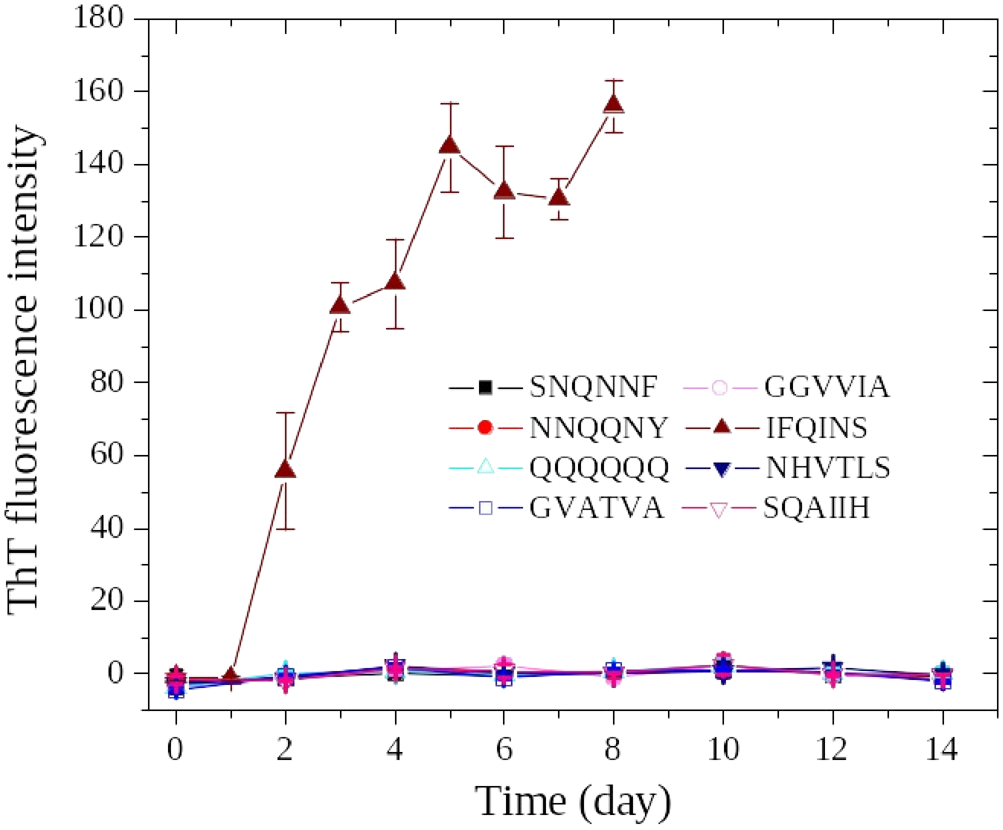

ThT fluorescence intensity
180
160
140
120
100
80
60
40
20
0
0
2
4
6
8
10
12
14
Time (day)
SNQNNF
GGVVIA
NNQQNY
IFQINS
QQQQQQ
NHVTLS
GVATVA
SQAIIH
