# Supplementary material for: Fibril-Forming Motifs Are Essential and Sufficient for the Fibrillization of Human Tau
Source: PLoS One. 2012 Jun 11;7(6):e38903. doi: 10.1371/journal.pone.0038903 (PMC3372541; doi:10.1371/journal.pone.0038903)
Supplement: Supplementary file 3 [file pone.0038903.s003.doc]

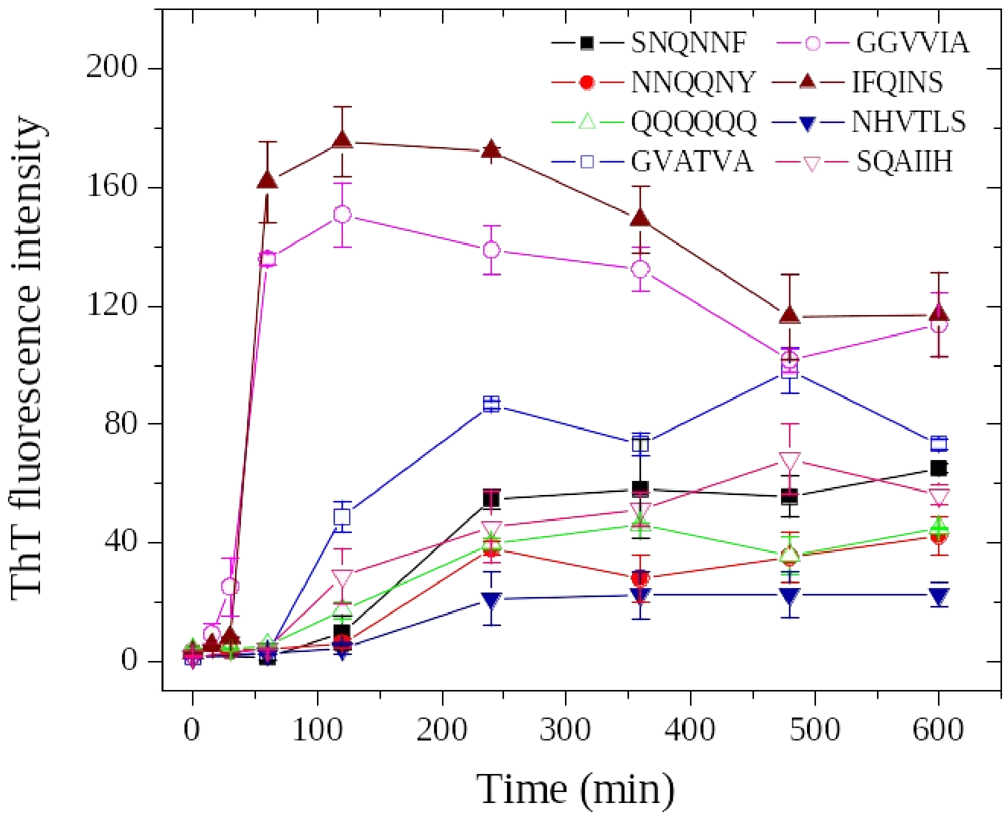

ThT fluorescence intensity
Time (min)
0
40
80
120
160
200
0
100
200
300
400
500
600
SNQNNF
NNQQNY
QQQQQQ
GVATVA
GGVVIA
IFQINS
NHVTLS
SQAIIH
